# Glycosylation Directs Targeting and Activation of Cystatin F from Intracellular and Extracellular Sources

**DOI:** 10.1111/j.1600-0854.2009.00881.x

**Published:** 2009-04

**Authors:** Jeff D Colbert, Anna Plechanovová, Colin Watts

**Affiliations:** 1 Division of Cell Biology and Immunology, College of Life Sciences, University of DundeeDundee DD1 5EH, UK; 2 Division of Gene Regulation and Expression, College of Life Sciences, University of DundeeDundee DD1 5EH, UK

**Keywords:** cathepsin C, cystatin, endocytosis/internalization, glycosylation, mannose 6-phosphate

## Abstract

Cystatin F is a cysteine protease inhibitor that is selectively expressed in immune cells and unlike other cystatin family members is targeted to a significant extent to intracellular compartments. Initially made as an inactive glycosylated disulfide-linked dimer, cystatin F is converted to an active monomer by proteolytic cleavage following transport to the endosomal/lysosomal system. This active form of cystatin F targets cathepsin C/DPPI and probably other cathepsins in immune cells. We show that efficient targeting of cystatin F to the endocytic pathway is dependent not on its unique dimeric conformation but rather on its oligosaccharide chains. We demonstrate the unusual addition of *N*-linked sugars to an Asn-X-Cys motif in cystatin F and provide evidence that the mannose 6-phosphate sorting machinery is used to divert cystatin F from the secretory pathway and to mediate its uptake from extracellular pools. These studies identify a function for the oligosaccharides on cystatin F and raise the possibility that cystatin F might regulate proteases *in trans*by secretion in an inactive form by one cell and subsequent internalization and activation by another cell.

The cystatins are a family of naturally occurring cysteine protease inhibitors comprising at least 11 members (1,2). Some are expressed in the cytosol, but the majority are made with signal sequences and enter the secretory pathway. Cystatins have been found in many different body fluids indicating that they have a role in protease regulation in the extracellular milieu. It is thought that cystatins are secreted into the extracellular space to inhibit proteases that may be inadvertently released from cells. However, it is not clear if this is their only or even their major function. Nonetheless, their importance is indicated by the severe phenotypes observed in the absence of some family members. For example, mice lacking cystatin E/M exhibit fatal abnormalities in development of the epidermis (3).

The cystatins are structurally well conserved and comprise a single α-helix associated with a 5-stranded β-sheet whose loops combine with residues from the N-terminus to form an ‘edge’ that engages the protease active site (4). Two cystatin/protease structures have been solved revealing in detail how this part of the ‘cystatin fold’ engages a cysteine protease (5,6). The primary targets of the cystatins are the papain-related C1 family of cysteine proteases that include enzymes such as cathepsins B, L and S. Some cystatins also inhibit the unrelated cysteine protease, asparagine endopeptidase, but through a distinct structural domain (7).

Cystatin F is an unusual member of the family in several respects. First, it is primarily expressed by immune cells (8,9) where it is often upregulated when these cells undergo differentiation or activation from quiescent precursors. Second, unlike all other family members, cystatin F is initially made as a disulfide-linked dimer (10). Moreover, this dimer is inactive as a protease inhibitor (11) because of mutual steric hindrance by one subunit of the other subunit’s ability to bind protease (12). This crucial finding strongly implies that the domain of action of cystatin F is intracellular because the secreted extracellular form is inactive. Consistent with this, a greater proportion of cystatin F is retained within cells compared with cystatin C (9,13). Third, unlike all other cystatins except cystatin E/M and in some instances rodent cystatin C (14,15), cystatin F is glycosylated carrying one (mouse) or two (human) *N*-linked oligosaccharides. Finally, the amino acids present in the protease-binding loops and N-terminal region of cystatin F are not homologous with those of other family members, indicating that cystatin F might bind distinct protease targets (8,9).

We recently strengthened the notion that cystatin F regulates intracellular rather than extracellular protease activity by showing that a principle target is cathepsin C (16). This enzyme is an important aminopeptidase that activates a variety of serine protease zymogens in the secretory granules of cytotoxic T lymphocytes (CTL), natural killer cells, mast cells and neutrophils (17–19). Although disruption of the cystatin F dimer is sufficient to generate an inhibitor of endopeptidases such as cathepsin L, a proteolytic cleavage event was required to convert inactive cystatin F into an active cathepsin C inhibitor. This cleavage event removes 15 N-terminal residues and also converts dimeric cystatin F into a monomeric form (16). This surprising finding also points to an intracellular domain of action.

How cystatin F is localized within the endocytic pathway when other family members are secreted has not been investigated. Several possibilities might account for its intracellular targeting. For example, the unique dimeric conformation of cystatin F may be important perhaps creating a recognition feature lacking in monomeric cystatins. Alternatively or additionally, one or both of the two canonical *N*-linked carbohydrate motifs may provide the necessary sorting signals. Third, because cystatin F is only 35% homologous to other family members, there may be other less obvious targeting motifs involved in its intracellular trafficking.

Here, we investigate how cystatin F is retained within cells by analysis of cystatin F mutants that either cannot dimerize or lack specific oligosaccharide chains. We show that cystatin F molecules that cannot dimerize are still efficiently targeted to the endosomal system. Moreover, a mutant of cystatin F lacking both canonical *N*-linked carbohydrate chains was also activated normally within the endocytic pathway. However, this ‘non-glycosylated’ variant of cystatin F retained sensitivity to treatment with *N*-glycosidase F (PNGase) because of carbohydrate addition on a cryptic noncanonical (Asn-X-Cys) *N*-glycosylation site. Either this site, or a neighboring canonical site, sufficed to specify sorting of cystatin F to lysosomal compartments. We further show that cystatin F made and secreted by one cell can be internalized and activated within a different cell through mannose 6-phosphate (M6P)-mediated targeting. Thus, immune cells expressing cystatin F may regulate *in trans*the protease activity of bystander cells.

## Results

### Cystatin F dimerization is not required for intracellular targeting

**Figure 1:**
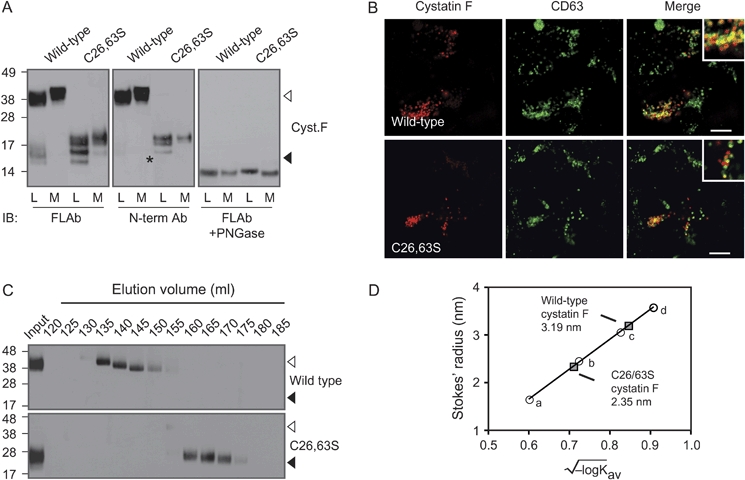
Cystatin F subcellular localization and secretion does not depend on dimer formation A) SDS–PAGE separated cell lysates (L) and culture medium (M) under nonreducing conditions from 293T cells transfected with wild-type or C26,63S human cystatin F. Western blots were probed with an antibody raised against full-length cystatin F (FL Ab) (left and right panels) or an N-terminal peptide (N-term Ab) (center panel). Right panel shows change in mobility following deglycosylation with PNGase under reducing conditions. Open and closed arrowheads denote dimeric and monomeric cystatin F, respectively. Asterisk denotes N-terminal processing of the C26,63S mutant. B) Immunofluorescence microscopy of 293T cells shown in (A) expressing wild-type or C26,63S cystatin F (red) and counterstained with an antibody to the lysosomal marker CD63 (green). Insets demonstrate areas of colocalization at high magnification. Bars, 10 μm. C) Western blot analysis following size exclusion chromatography. Nonreducing SDS–PAGE gel analysis of equal sized aliquots of column fractions obtained following chromatography of wild-type (top panel) or C26,63S (bottom panel) cystatin F. D) The Stokes’ radius of wild-type (3.19 nm) and C26,63S (2.35 nm) cystatin F were calculated following size exclusion chromatography. Values were interpolated from the calibration curve obtained by the linear fitting of Stokes’ radius versus (−log *K*_av_)^1/2^ for a series of proteins with known Stokes’ radii, as described. Protein calibration was obtained using ribonuclease A (a), carbonic anhydrase (b), ovalbumin (c) and conalbumin (d).

To investigate the trafficking of cystatin F, we generated various mutant forms and analyzed these initially in the human kidney epithelial cell line 293T, which does not express cystatin F. The cells were transfected, and after 48 h, the relative amount of cystatin F in cell lysates (L) and in the culture medium (M) was assessed. Expression of wild-type cystatin F leads to significant secretion of the protein into the culture medium as we and others have previously reported (10,13,16). Nonetheless, a significant amount of cystatin F was also retained in 293T cells and was partially converted to the monomeric form (Figure 1A, left panel). Cystatin F recovered from the culture medium was dimeric, whereas cellular cystatin F comprised a mixture of dimeric and monomeric protein (Figure 1A, left panel). Furthermore, while the dimeric form reacted with antibodies raised against the N-terminus of cystatin F the monomeric form did not, indicating intracellular processing had occurred as we recently reported (Figure 1A, center panel) (16). As shown previously, heterogeneous *N*-glycosylation accounted for the resolution of distinct forms of cystatin F on SDS–PAGE (9). This is highlighted by the formation of a single form following treatment with PNGase (Figure 1A, right panel).

Cystatin F dimerizes through two disulfide bridges involving Cys26 on one subunit and Cys63 on the other (12). We first asked whether intracellular retention of cystatin F was dependent on dimerization by transfecting 293T cells with a cysteine to serine double mutant (C26,63S cystatin F). The expressed protein was monomeric, as expected. Moreover, a similar proportion was retained within the cells when compared with wild-type cystatin F (Figure 1A, left panel), indicating that dimerization is not necessary to divert cystatin F from the secretory to the endocytic pathway. Monomeric cystatin F appeared to be glycosylated normally as judged by its mobility and sensitivity to PNGase (Figure 1A). In addition, a proportion of monomeric cystatin F was N-terminally processed as judged by the reactivity of the fastest migrating form with antibody raised against whole cystatin F but not with the N-terminal-specific N1 antibody (Figure 1A, asterisk).

To confirm targeting to the endocytic pathway, transfected cells were fixed and stained with antibodies against cystatin F and a marker of the late endosomal/lysosomal compartment (CD63). As shown in Figure 1B, cystatin F partially colocalized with CD63 indicating lysosomal localization. Moreover, C26,63S cystatin F was also colocalized with lysosomal markers.

Although covalent dimer formation was circumvented by substitution of the two cysteines involved in disulfide bond formation with serine, noncovalent dimer formation was still plausible. In other words, dimerization of C26,63S through noncovalent interaction may account for its lysosomal localization and secretion. To address this, wild-type and C26,63S cystatin F were subject to size exclusion chromatography on a column calibrated with standard proteins of known molecular weight and stokes’ radii. Elution of cystatin F was initially followed by absorption (280nm) of the eluted fractions and then confirmed by western blot (Figure 1C). As expected, wild-type cystatin F eluted as a single species and had a calculated stokes’ radius of 3.19 nm (Figure 1C, top panel). Likewise, C26,63S cystatin F eluted as a single species but with a smaller calculated stokes’ radius of 2.35 nm (Figure1C,D). SDS gel analysis of individual column fractions confirmed that C26,63S cystatin F was included in the column to a much greater extent than wild-type protein. Together, these data indicate that while cystatin F dimerizes, is glycosylated and a proportion is targeted to the endocytic pathway in 293T cells, dimerization is not necessary for these post-translational modifications or for its targeting.

### Noncanonical Asn-linked glycosylation can occur on cystatin F

**Figure 2:**
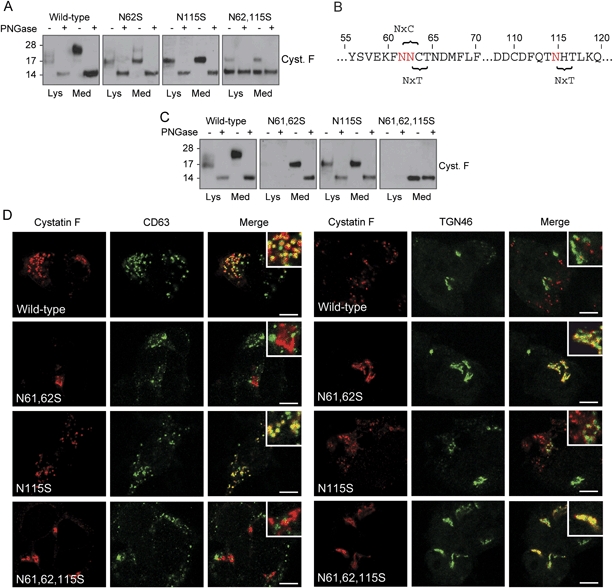
Mutagenesis of consensus sequences for *N*-linked glycosylation reveals an atypical glycosylation site A) Reducing SDS–PAGE analysis of mutants of one (N62S or N115S) or both (N62,115S) *N*-linked glycosylation sequences. Remaining glycosylation of cystatin F (Cyst. F) is revealed by comparing untreated samples (−) to PNGase treatment (+). (B) Human cystatin F amino acid residues 61–63 (NXC) represent a potential atypical *N*-linked glycosylation site. (C) Reducing SDS–PAGE comparing glycosylation of double (N61,62S) and triple (N61,62,115S) Asn mutants in the presence (+) or absence (−) of PNGase. Representative cell lysates (Lys) and culture medium (Med) are shown. (D) Immunofluorescence microscopy following transfection of 293T cells (as in C) with antibodies raised against full-length cystatin F (red) and markers of lysosomes (CD63) or the *trans*-Golgi network (TGN46) (green). Insets reveal areas of costaining. Bars, 10 μm.

We next assessed the possibility that glycosylation of cystatin F was important for targeting the endosomal/lysosomal system. In human cystatin F asparagines 62 and 115 carry carbohydrate chains, whereas murine cystatin F carries a single *N*-linked sugar at a site homologous to Asn62 in humans. We generated mutant forms of human cystatin F where one or both Asn residues were altered to Ser (N62S, N115S and N62,115S) and transfected these into 293T cells. Culture medium and cell lysates were then analyzed for the presence of cystatin F and digested with PNGase to assess its glycosylation status. Similar to wild-type cystatin F, all mutant forms of cystatin F migrated as a single band following treatment with PNGase. Puzzling, however, the gel mobility of the N62S mutant in the absence of PNGase digestion was similar to that of wild type (Figure 2A). Even more surprising is that the double mutant N62,115S, which we predicted would not be glycosylated, migrated as two species, one of which retained sensitivity to digestion with PNGase. Because N62 and N115 are the only Asn residues found in a conventional Asn-X-Thr/Ser glycosylation consensus sequence, these results pointed to the existence of an unusual glycosylation site on cystatin F. Glycosylation of Asn residues in the context an atypical Asn-X-Cys sequence has previously been observed, for example in the recombinant epidermal growth factor receptor, the T-cell activation antigen CD69 and a few other proteins (20,21). As shown in Figure 2B, Asn61 of cystatin F also occurs in this context. We therefore tested the hypothesis that mutagenesis of Asn62 might reveal a cryptic noncanonical glycosylation site at Asn61. Consistent with this possibility, the double mutant N61,62S now expressed a single PNGase-sensitive product, and the triple mutant N61,62,115S was fully resistant to the action of PNGase (Figure 2C). Thus, the Asn^61^-X-Cys^63^ sequence in cystatin F can be used by the glycosylation machinery.

### Glycosylation of either Asn61 or Asn62 is required for cystatin F targeting to endosomes

Analysis of the trafficking of the Asn61 mutant produced another important result. Whereas the N62,115S mutant was still targeted to intracellular compartments (data not shown), the N61,62S mutant and the triple mutant were largely absent and found mostly in the medium (Figure2C). Consistent with this, we could not detect colocalization of the N61,62S or N61,62,115S mutant with markers of the lysosomal pathway (Figure 2D). Moreover, in contrast to cells expressing wild-type and N115S mutants, cathepsin C activity was not inhibited (Figure S1). Instead, any cystatin F remaining within these cells colocalized with the markers of the *trans*-Golgi network indicative of protein destined for secretion (Figure 2D). These results indicate that glycosylation of cystatin F is important for its intracellular targeting and that *N*-linked glycans on either Asn61 or Asn62 are required. In contrast, glycosylation of Asn115 does not appear to be involved in the intracellular targeting of cystatin F.

### Mannose 6-phosphorylation is used to target cystatin F to endosomes

**Figure 3:**
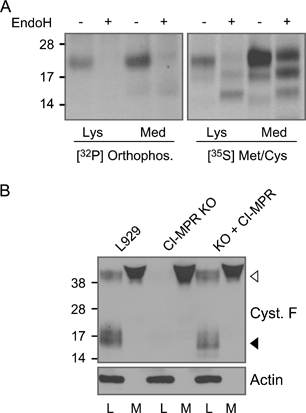
Cellular retention of cystatin F requires the CI-MPR A) 293T cells transfected with wild-type cystatin F were radiolabeled with [^32^P] orthophosphate (left panel) or [^35^S] methionine/cysteine (right panel). Labeled cystatin F in postnuclear lysates (Lys) and culture medium (Med) were immunoprecipitated and either left untreated (−) or treated with EndoH (+) followed by SDS–PAGE under reducing conditions. B) Retrovirus containing wild-type human cystatin F was used to infect L929 cells, L cells lacking the CI-MPR (CI-MPR KO) or the CI-MPR KO reconstituted with the CI-MPR (KO+CI-MPR). Cystatin F in cell lysates (L) and culture medium (M) was detected by western blot using the FL Ab following SDS–PAGE under nonreducing conditions. Open and closed arrowheads denote dimeric and monomeric cystatin F, respectively. Actin staining is used to show equivalent protein loaded.

Many lysosomal hydrolases carry *N*-linked glycans that become modified with M6P and use the cation-dependent or -independent M6P receptors (CD-MPR or CI-MPR, respectively) for targeting the endocytic pathway (22). We asked whether cystatin F might also utilize the M6P system to reach endosomes and lysosomes. First, we looked for evidence that one or more of the oligosaccharides on cystatin F were phosphorylated. As shown in Figure 3A, cystatin F became labeled with [^32^P] orthophosphate in 293T cells, and this label was removed by treatment with endo-β-*N*-acetylglucosaminindase H (EndoH), suggesting phosphorylation occurred on high mannose glycans. In contrast, [^35^S]-labeled cystatin F migrated faster following treatment with EndoH, but the label was not removed. Next, we infected mouse fibroblasts, including those lacking the CI-MPR, with a retrovirus expressing wild-type cystatin F. Whereas L929 cells, which express wild-type levels of both MPR, retained a proportion of cystatin F and converted it to the monomeric form, no intracellular cystatin F could be detected in L cells lacking the CI-MPRs (Figure 3B). Cystatin F retention and N-terminal processing were restored in knockout L cells reconstituted with the CI-MPR (Figure 3B).

### Cellular acquisition of exogenous cystatin F

The itinerary of the CI-MPR is known to include the cell surface and many studies have demonstrated its ability to mediate internalization of extracellular M6P-tagged enzymes. Our finding that intracellular targeting of cystatin F could use the M6P system raised the possibility that secreted cystatin F might enter cells using the same system. More generally, the internalization and subsequent activation of inactive dimeric cystatin F would in principle allow proteases to be regulated *in trans*, that is in cells not expressing cystatin F.

**Figure 4:**
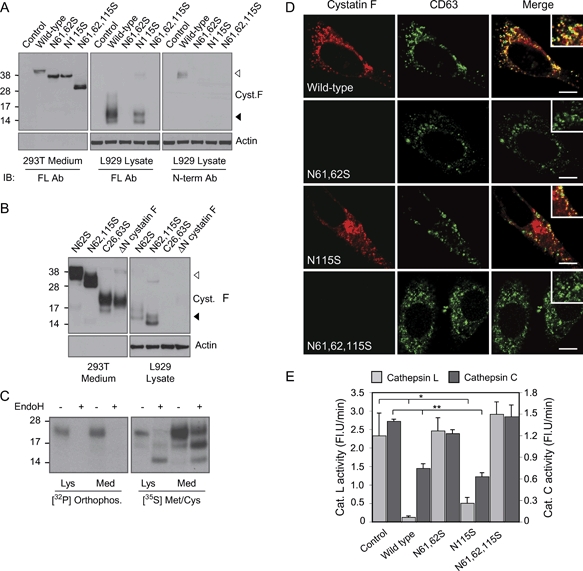
Secreted cystatin F can be internalized in a carbohydrate-dependent manner A) Equal concentrations of secreted wild-type and glycosylation mutant cystatin F (Cyst. F) produced by 293T cells (left panel) were cultured with L929 cells for 48 h. L929 cell lysates were separated by SDS–PAGE under nonreducing conditions with membranes immunoblotting for internalized cystatin F using the indicated antibody (center and right panel). B) Equal concentrations of secreted glycosylation mutants (N62S and N62,115S) and mutants rendering cystatin F monomeric (C26,63S and ΔN cystatin F) produced by 293T cells (left panel) were cultured with L929 cells as described in (A) and were stained with the FL Ab (right panel). Cystatin F dimeric species (open triangles) and monomeric species (closed triangles) are shown. Actin staining indicates equal protein loading. C) 293T cells transfected with C26,63S cystatin F were radiolabeled with [^32^P] orthophosphate (left panel) or [^35^S] methionine/cysteine (right panel). Labeled cystatin F in postnuclear lysates (Lys) and in the culture medium (Med) were immunoprecipitated using the cystatin F polyclonal antibody and either left untreated (−) or were treated with EndoH (+) followed by SDS–PAGE under reducing conditions. D) Immunofluorescent labeling of internalized cystatin F (red) in L929 cells and the lysosomal marker CD63 (green). Insets demonstrate areas of colocalization at high magnification. Bars, 10 μm. (E) Cathepsin activities in 293T cells (cathepsin C; light gray) and L929 cells (cathepsin L; dark gray) exposed to cystatin F-conditioned medium. Data represent the mean rate of more than three independent experiments. Error bars indicate the SEM. Significant differences of *p < 0.02 and **p < 0.005.

We first asked whether cystatin F secreted by 293T cells could be taken up by other cells and if so whether internalization was dependent on cystatin F glycosylation. We collected the culture medium from 293T cells transfected with the various *N*-glycosylation mutants of cystatin F described above (Figure 4A, left panel) and incubated these conditioned media with L929 cell monolayers. After 48 h, the recipient cells were tested for internalization and processing of cystatin F. L929 cells not only acquired wild-type cystatin F but also converted the inactive dimer to the monomeric form (Figure 4A, right panel). Although N115S cystatin F was also taken up by L929 cells, the N61,62S and N61,62,115S forms of cystatin F were not (Figure 4A). The internalization of cystatin F glycan mutants N62S or N62,115S further confirmed the requirement of glycosylation at one of these important residues. Surprisingly, monomeric cystatin F (C26,63S) and a mutant mimicking the N-terminally processed cystatin (ΔN cystatin F) (16) failed to be internalized (Figure 4B). As shown in Figure4C, C26,63S cystatin F, like wild-type cystatin F, still acquired EndoH-sensitive phosphate residues indicating that monomeric cystatin F was also modified with M6P (Figure 4C). These data suggest that in addition to glycosylation of N61 or N62, a dimeric and presumably dually phosphorylated protein must be present. We confirmed these results by immunofluorescence microscopy. Wild-type and N115S cystatin F, but not N61,62S or N61,62,115S cystatin F, were taken up and became colocalized with CD63 (Figure 4D). Importantly, internalized cystatin F became active as a protease inhibitor within the recipient cells because endogenous cathepsin L and cathepsin C activity was suppressed. In contrast, there was no inhibition of these proteases in cells incubated with N61,62S or N61,62,115S cystatin F (Figure 4E).

**Figure 5:**
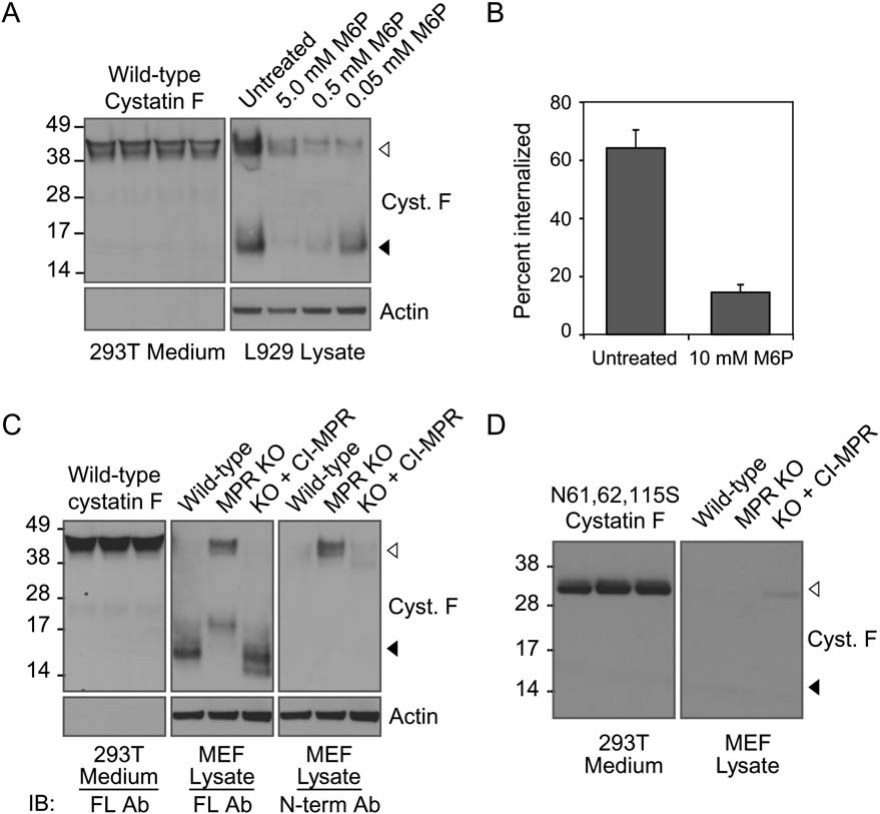
Internalization and activation of cystatin F is dependent on the CI-MPR A) L929 cells cultured in media conditioned by cystatin F-transfected 293T cells (represented in the left panel) in the absence or presence of decreasing concentration of free M6P (5.0–0.05 mmfinal). B) [^125^I]-labeled recombinant human cystatin F was cultured with L929 cells in the presence or absence of free M6P for 30 min at 37°C. The percent of cystatin F internalized was measured by comparing surface bound [^125^I]-labeled cystatin F at 0 min with internalized cystatin F at 30 min as described in the *Materials and Methods*. C) Cystatin F-containing medium (left panel) was cultured with wild-type MEF, MPR knockout MEF (MPR KO) or in fibroblasts reconstituted with only the CI-MPR. Left and center panels display gels probed with the FL Ab. Right panel shows MEF lysates probed with the N-term Ab. D) Unglycosylated cystatin F (N61,62,115S)-containing medium (left panel) was cultured with either wild-type MEFs, MPR KO MEFs or in reconstituted MEFs (right panel). Western blots were probed with the FL Ab. All gels were run under nonreducing conditions. Open and closed arrowheads denote dimeric and monomeric cystatin F, respectively. Actin staining is used to show equivalent total protein loaded.

To establish whether the glycosylation-dependent internalization of cystatin F utilized the M6P system, we repeated the internalization experiment in the presence of increasing concentrations of M6P. As shown in Figure 5A, accumulation of monomeric cystatin F by L929 cells was significantly blocked in the presence of 0.5 mmM6P and almost completely by higher concentrations of M6P (Figures 5A,B). This blockade was specific for M6P as pretreatment with mannose or fucose failed to inhibit cystatin F internalization (Figure S2). However, there was a small fraction of dimeric cystatin F associated at these concentrations of M6P. We also tested mouse embryonic fibroblasts (MEF) isolated from either wild-type or MPR knockout mice. Whereas monomeric cystatin F accumulated in wild-type MEF and in knockout MEF reconstituted with the CI-MPR, knockout MEF themselves accumulated much less monomer (Figure 5C). As in the presence of M6P, a fraction of dimeric cystatin F continued to associate with these cells as did some faster migrating cystatin F. However, this cystatin F did not co-migrate with the monomer taken up by wild-type cells, although it appeared to be N-terminally processed (Figure 5C, center and right panel). To examine whether the residual cystatin F association with cells devoid of MPR was carbohydrate dependent, we exposed wild-type and knockout MEFs to unglycosylated cystatin F. As shown in Figure5D, internalization of N61,62,115S cystatin F was completely blocked irrespective of the presence or absence of the MPR. These results show that internalization and activation of cystatin F is at least partially dependent on the MPR system and is completely dependent on glycosylation of cystatin F.

### Cystatin F production and internalization by immune cells

**Figure 6:**
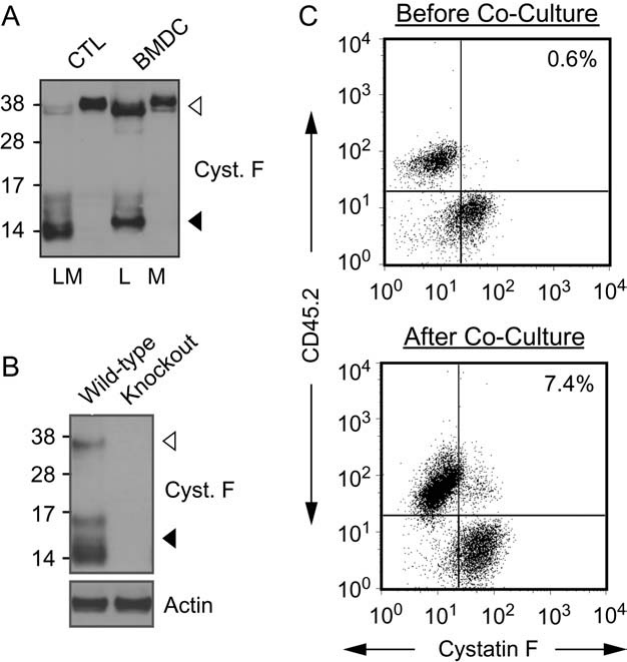
Internalization and activation of cystatin F in primary cells A) Lysates (L) and medium (M) from cultured primary murine CD8^+^ T lymphocytes (CTL) and dendritic cells (BMDC) isolated from spleen and bone marrow, respectively. B) Lysates from activated CD45.1^+^ (wild type) and CD45.2^+^ (knockout) CTLs were separated by nonreducing SDS–PAGE and immunoblotted for cystatin F (Cyst. F). Arrowheads denote dimeric (open) and monomeric (closed) cystatin F. Actin staining is used to confirm equivalent protein loading. C) CD45.1^+^ wild-type splenocytes were cocultured with CD45.2^+^ cystatin F knockout splenocytes for 48 h. Internalization of cystatin F into CD45.2^+^ cells was determined by intracellular immunostaining (after coculture). Numbers represent percentage of CD45.2^+^ cells staining for cystatin F before and after coculture. FACS data are representative of at least three independent experiments.

Finally, we asked whether secretion of cystatin F by one cell and internalization by another could be demonstrated in primary immune cells. We found that significant amounts of dimeric cystatin F were secreted from primary CD8^+^ T lymphocytes and bone marrow-derived dendritic cells (BMDC). As expected, the cell lysates also contained cystatin F, much of which was converted to the active monomeric form (Figure 6A). We recently generated cystatin F-deficient mice that are currently being analyzed. These mice were used to generate a population of cystatin F-negative CD8^+^ T cells by *in vitro*expansion. We incubated these cells with an equal number of wild-type CD8^+^ T cells and asked whether cystatin F was acquired by the null cells. We used the CD45 marker and fluorescence-activated cell sorter (FACS) analysis to distinguish wild type (CD45.1^+^) from cystatin F null (CD45.2^+^) cells (Figure 6B, top panel). As shown in Figure 6C, a small proportion of CD45.2^+^ cells became positive for cystatin F during the course of the experiment demonstrating that the protease inhibitor had been taken up by the null cells (Figure 6C, bottom panel). This important result indicates that one cell may influence the protease activity of another through the agency of cystatin F.

We sought to determine whether internalized cystatin F was active in recipient primary T cells. However, it was not feasible to analyze proteolytic activity in the relatively small number of cells that had acquired cystatin F because their identification necessitated fixation and permeabilization before FACS analysis. To establish that protease activity in primary T cells can be attenuated by exogenous cystatin F, we cultured cystatin F knockout splenocytes with recombinant cystatin F for 48 h and followed its internalization by intracellular FACS analysis and microscopy. A parallel culture was then lysed and cathepsin C protease activity was analyzed in the cystatin F-treated and untreated populations. Internalization of cystatin F under these conditions resulted in its proper localization (Figure S4A,B). Moreover, although not all T cells acquired cystatin F, cathepsin C activity in the bulk population was substantially reduced in the cystatin F-exposed cells (Figure S4C), demonstrating that internalized cystatin F was active. This result further indicates that cystatin F secreted by one cell may attenuate protease activity in another cell.

## Discussion

The mechanism of action of cystatin F appears to constitute a new paradigm for regulation of protease activity in the endocytic pathway. Following biosynthesis, its activity is quenched because of its unique dimeric conformation (12). Some of this form is secreted from cells but a substantial proportion is targeted to the endocytic pathway where proteolysis converts the dimer to active monomers. In immune cells, this form inhibits cathepsin C/DPPI and probably other cathepsins (16). The existence of a protease-activated protease inhibitor clearly suggests a novel mechanism for feedback attenuation of protease activity in the endocytic pathway. How cystatin F is targeted to the endocytic pathway is an important and unresolved issue that we investigated here.

Most cystatins made with signal sequences (type II cystatins) are secreted from cells, suggesting that their primary function is to inactivate potentially dangerous proteases that have been secreted or lost from cells into the extracellular space thereby protecting from inflammatory reactions associated with aberrant release of these proteases (23–25). While cathepsins have been shown to be secreted from cells and have been shown to exhibit protease activity at neutral pH (26–28), their inhibition by secreted cystatins has not been demonstrated. Cystatin F has also been shown to be secreted (9); however, recent data suggest that the primary targets for cystatin F are intracellular. First, the relative concentration of cystatin F within cells compared with that which is secreted is much higher than for the other type II family members (13). Second, our recent findings suggest that one of the true physiological targets of cystatin F is cathepsin C, which is found in the endocytic compartment (16). Inhibition of this aminopeptidase requires that cystatin F undergoes an N-terminal proteolytic processing step occurring in intracellular compartments where protease activity and its cathepsin targets are likely to reside (16). Third, cystatin F is secreted as an inactive disulfide-linked dimer, and to date, the active monomeric form has only been observed intracellularly (11,16). Together, these data suggest that cystatin F inhibitory activity is restricted to the endocytic compartment.

The mechanism by which cystatin F is retained within cells and matured in the endocytic compartment is addressed here. We demonstrate that dimerization is not necessary for intracellular cystatin F to be directed to the lysosomal pathway but rather a carbohydrate-dependent sorting mechanism operates. Our findings provide an explanation for why cystatin F is glycosylated when almost all other family members are not. Cystatin M/E is also glycosylated and is found in the lamellar granules of the cells of the stratum granulosum of the skin as well as in extracellular locations (29). Whether localization of cystatin M/E in these granules also depends on its glycosylation is not known.

To our knowledge, cystatin F is the first lysosomal protease inhibitor shown to be M6P modified. This appears to be integral for proper targeting to the lysosomal compartment as judged by the failure of cells lacking MPRs to retain cystatin F. An earlier report identified cystatin F as one of several secreted proteins recovered from an MPR column, although it was not clear if the association was direct or indirect, for example through a binding partner modified with M6P (30). Our data indicate that binding to the MPR column was most likely direct. Studies in cells derived from mice lacking both 46 and 300 kD MPRs demonstrate that some cell types have additional mechanisms for intracellular targeting of lysosomal hydrolases (31,32). It is possible that cystatin F also utilizes such mechanisms depending on cell type.

Human cystatin F carries two *N*-linked oligosaccharides at Asn62 and 115, whereas murine cystatin F lacks the second sugar. Consistent with conservation of glycosylation at position 62 in mammalian cystatins (Figure S3), we show that this sugar carries targeting information, whereas the Asn115-linked sugar does not. Surprisingly, elimination of both Asn62 and 115 revealed a cryptic *N*-linked glycosylation site at Asn61, which utilized the noncanonical motif Asn-X-Cys-Ser/Thr and which sufficed to target cystatin F correctly in the absence of the canonical Asn62-linked sugar. The Asn61 residue is conserved in all cystatin F sequences deposited thus far (Figure S3). Other proteins have been shown to direct glycans at sites containing cysteine rather than serine or threonine at the third position. Like cystatin F, several include atypical sequences where a serine or threonine residue at the fourth position in conjunction with a cysteine at the third position (NXCS/T) is present. These include the immune regulator CD69 and human protein C (20,21,33)**.**Whether Asn61 and Asn62 are both modified in wild-type cystatin F is not yet clear. Because the cysteine residue (C63) in the Asn-X-Cys motif is involved in the dimerizing disulfide bond, presumably recognition of the motif and addition of core oligosaccharide to Asn61 must occur before dimerization and loss of the free thiol. However, this has not been formally demonstrated.

High concentrations of reducing agents are required to reduce the cystatin F dimer making a proteolytic mechanism for monomerization more likely and linking cystatin F activation to endosomal proteolytic activity rather than reducing potential (11,16). Based on the crystal structure of cystatin F, we suggested earlier that an additional function for the oligosaccharide at Asn62 (and perhaps Asn61) may be to shield the disulfide bonds linking the monomers (12). However, as cystatin F lacking oligosaccharides at Asn61 and Asn62 is secreted, this remains a hypothesis because we are unable to assess whether this dimer is more sensitive to reduction *in vivo*.

Sequestration of cystatin F from the secretory pathway by an M6P/MPR mechanism raised the possibility that secreted dimeric cystatin F might be taken up into cells using the same system, as first described by Neufeld and colleagues for lysosomal enzymes (34). We found that different cell lines and primary cells were able to internalize inactive dimeric cystatin F and convert it to its active monomeric form. Moreover, this internalization and conversion was inhibited by free M6P sugar and was reduced in cells lacking MPRs. Importantly, we could demonstrate suppression of protease activity in the recipient cell as well as exchange of cystatin F between immune cells. We noticed that secreted monomeric cystatin F was not internalized although, like wild-type cystatin F, it was phosphorylated on high mannose glycans and was targeted to lysosomes during biosynthesis. Conceivably, the increased avidity provided by two M6P modified sugars in the dimer for the CI-MPR accounts for this observation (35). It is also important to note that soluble monomeric M6P could not completely suppress cystatin F internalization into L929 cells, and consistent with this, some internalization still occurred in cells lacking MPRs. However, additional mechanisms for targeting secreted lysosomal enzymes exist such as the multiligand receptor megalin (36,37); carbohydrate-specific-binding lectins such as the galactose-, fucose- and mannose-binding receptors (38–40) and glycosaminoglycan (GAG)-binding receptors such as CD44 and the hyaluronic acid receptors (41,42), among others. It is possible that one or more of these mechanisms contribute to cystatin F targeting and endocytic uptake. Indeed, a recent report shows that human cystatin C, which is monomeric and unglycosylated (43), can be taken up by cells via an unknown mechanism (44). However, the role of carbohydrates associated with cystatin F is highlighted by the fact that unglycosylated cystatin F was not internalized by target cells. Furthermore, in the absence of M6P targeting, most endocytosed cell-associated cystatin F remained in the dimeric form, suggesting that efficient transport to the compartment where proteolytic conversion occurred required the M6P/MPR system.

These results raise the possibility that immune cells secreting cystatin F may be able to regulate protease activity in neighboring cells that either do not express cystatin F or express insufficient levels. Cystatin F expressing primary cell populations such as CD8^+^ T cells often show heterogeneous levels of expression. Therefore, secretion and internalization of cystatin F in the closely confined *in vivo*milieu may even out these differences in expression. Cystatin F may be transferred efficiently from one cell to another not only because of its M6P tag but also because, unlike other cystatins found in the external milieu, it is unable to bind protease targets and therefore will not be sequestered by extracellular cysteine proteases. Several precedents exist for *in vivo*acquisition of lysosomal enzymes by one cell type following secretion by another. For example, cathepsin L-deficient thymocytes acquired this enzyme from surrounding epithelial cells rescuing a defect in antigen presentation and development of natural killer T cells (45). In another example, procathepsin B was taken up *in vivo*from the glomerular filtrate into kidney proximal tubule cells and reconstituted a cathepsin B deficiency (36). Although we have not yet demonstrated transfer of cystatin F between cells *in vivo*, our results clearly indicate that this may take place. Conceivably, given that cathepsin C is one of the targets of cystatin F, this may be a way of attenuating granule protease-driven inflammatory reactions.

**Figure 7:**
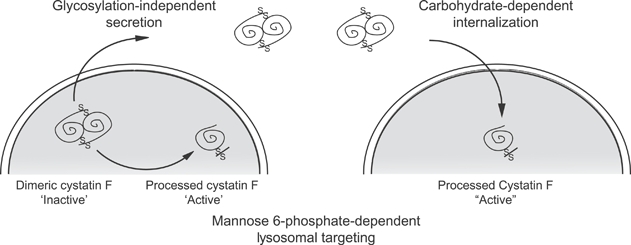
Proposed model describing the requirement of cystatin F glycosylation for intracellular targeting and internalization Cystatin F is synthesized as an inactive glycosylated disulfide-linked dimer. M6P modified glycans direct cystatin F to lysosomes where N-terminal proteolysis induces its activation (processed cystatin F). A proportion of inactive dimeric cystatin F is secreted where it binds membrane receptors, such as the CI-MPR, on adjacent cells. Internalization of cystatin F leads to a M6P-dependent lysosomal localization where N-terminal processing results in its activation.

In summary, our study sheds light on the unusual features of the cystatin F molecule. Glycosylation and more specifically M6P modification serve to target it to the endocytic pathway during biosynthesis, and for a proportion of molecules, by endocytosis from the external medium (Figure 7). In the event when Asn62 is not modified, a ‘backup’ noncanonical *N*-linked M6P-modified sugar can be added to Asn61. Although the dimeric form is not crucial for intracellular targeting, it appears to be necessary for endocytic uptake. Moreover, dimerization ensures the latency of the inhibitor, which only becomes active following activation by proteases. The possibility that cystatin F is activated by the same protease systems that it inhibits is currently under investigation.

## Materials and Methods

### Chemical reagents

Cell culture media were purchased from Gibco unless otherwise indicated. H-Gly-Phe-AMC and Z-Phe-Arg-AMC were purchased from Bachem. PNGase and EndoH were purchased from Roche. Rabbit polyclonal antibodies directed against full-length human cystatin F (FL Ab) or against an N-terminal peptide (aa20-35; N-term Ab) have been described previously (16). Mouse anti-CD63 was purchased from Abcam. Sheep anti-TGN46 antibody was a kind gift from Dr S. Ponnambalam (University of Leeds, UK). Mouse monoclonal actin antibody was purchased from Sigma-Aldrich. All secondary antibodies used for western analysis were purchased from Jackson Laboratories. Antigen-presenting cell (APC)-conjugated CD45.2, CD8 and CD11c were purchased from eBioscience. Quik Change Site-Directed Mutagenesis kit was purchased from Stratagene. Protein standards for determination of stokes’ radii were obtained from GE Healthcare.

### Cell culture

The cell lines 293T and L929 were cultured in DMEM supplemented with 10% fetal calf serum (FCS), 2 mml- glutamine, 100 U/mL penicillin and 100 μg/mL streptomycin. L cells devoid of the CI-MPR (CI-MPR KO), and those reconstituted with the CI-MPR (KO + CI-MPR) were kindly provided by T. Braulke (University Hospital Hamburg, Germany) and were cultured in the same medium as L929 cells. Wild-type MEF, MPR knockout fibroblasts and those reconstituted with the human CI-MPR were generous gifts from R. Pohlmann (Westfälische Wilhelms-Universität Münster, Germany) and B. Hoflack (Biotechnological Center, Technical University of Dresden, Germany) and were cultured as described (46). Dendritic cells and CD8^+^ T lymphocytes were cultured from the bone marrow and spleen, respectively, of C57BL/6 mice as described previously (16,47). C57Bl6-Ly5.1^+^ (CD45.1^+^) mice were a kind gift from P. Crocker (University of Dundee, UK). Cystatin F knockout mice were generated under contract by Artemis Pharmaceuticals GmbH and will be fully described elsewhere.

### Site-directed mutagenesis and deglycosylation of cystatin F

Mutagenesis was performed using plasmids encoding full-length human cystatin F cDNA in a pcDNA-DHFR expression vector as described previously (16). Cysteine mutants were constructed at amino acids C26 and C63 by exchanging the Cys to Ser as described previously (16). Glycosylation mutants were constructed at amino acids N61, N62, N115S or combinations therein by exchanging Asn to Ser using the following primers: N62S, CAGTGTTGAAAAGTTCAACTCCTGCACGAACGACATGTTCTTGTTC and GAACAAGAACATGTCGTTCGTGCAGGAGTTGAACTTTTCAACACTG; N61,62S, CAGTGTTGAAAAGTTCAGCTCCTGCACGAACGACATGTTCTTGTTC and GAACAAGAACATGTCGTTCGTGCAGGAGCTGAACTTTTCAACACTG and N115S, GACTTCCAAACCAGCCACACCTTGAAGCAGACTCTGAGCTGCTAC and GTAGCAGCTCAGAGTCTGCTTCAAGGTGTGGCTGGTTTGGAAGTC. Underline denotes substituted residues. All mutagenesis was performed using the Quik Change Site-Directed Mutagenesis kit. Glycosylation of cystatin F was assessed by treating cell lysates, conditioned medium or immunoprecipitated cystatin F with PNGase to remove the entire glycan or EndoH to selectively remove high mannose forms of the glycans according to manufacturer’s guidelines.

### Transfection, transduction and protease assays

293T cells were transfected with plasmids containing either full-length (wild type) or mutant cystatin F following manufacturer’s instructions (Lipofectamine 2000; Invitrogen). Human cystatin F was transduced into murine L929 and L cells using the retroviral vector pBMN-I-GFP (Addgene, originally from G. Nolan, Stanford) as described previously (16). Transduced and transfected cells were left in culture for 48 h after which cystatin F-enriched medium and cells were separated by centrifugation. Cells were washed and lysed in buffer containing 100 mmcitrate, pH 5.4, 150 mmNaCl, 2 mmMgCl_2_ and 1% Triton-X-100. Culture media and cell lysates were analyzed for cystatin F expression by western blot using antibodies directed against the whole protein (FL Ab) unless otherwise noted. Protein concentrations in cell lysates were quantified by Bio-Rad Protein Assay (Bio-Rad). Equivalent concentrations of cystatin F in culture medium were determined by western blot and normalized before internalization assays, as described below.

Cathepsin activities were determined from 293T or L929 cell lysates following transfection or incubation with cystatin F-enriched media, as described below. Ten micrograms of postnuclear cell lysates was assayed using either 50 mmH-Gly-Phe-AMC (cathepsin C) or 40 mmZ-Phe-Arg-AMC (cathepsin L) in assay buffer containing 150 mmNaCl, 2 mmethylenediaminetetraacetic acid, 5 mmDTT and 100 mmcitrate, pH 5.5. All fluorometric assays were performed on a FLUOstar Optima Fluorimeter (BMG) reading excitation at 360 nm and emission at 460 nm. Cathepsin activity in 293T cells (cathepsin C) and L929 cells (cathepsin L) are expressed as the rate of AMC release over time.

### Size exclusion chromatography

Stokes’ radius determination of wild-type dimeric cystatin F, C26,63S cystatin F and standard proteins were performed by size exclusion chromatography using an Amersham HiLoad 26/60 Superdex 75 column (Amersham Pharmacia). The column was pre-equilibrated in 50 mmphosphate buffer and 0.15 M NaCl, pH 7.2. Protein was loaded in 0.8 mL of equilibration buffer in detergent-free conditions at a flow rate of 0.5 mL/min at room temperature on the ÄKTA fast protein liquid chromatography system (Amersham Pharmacia) collecting 1 mL fractions. The elution volumes of cystatin F and standard proteins (*V*_e_) were converted into *K*_av_ using the following equation; *K*_av_ = (*V*_e_ − *V*_o_)/(*V*_c_ − *V*_o_), where *V*_o_ is the void volume as determined by blue dextran and *V*_c_ is the calculated geometric column volume (*V*_c_ = *r*^2^∏*L*). The Stokes’ radius of cystatin F was determined using a linear calibration plot of stokes’ radii versus (−log *K*_av_)^1/2^(48) obtained from standard proteins with known molecular weight and stokes’ radii; ribonuclease A (13.7 kDa, 1.64 nm), carbonic anhydrase (29k Da, 2.43 nm), ovalbumin (43 kDa, 3.05 nm) and conalbumin (75 kDa, 3.57 nm as calculated).

### Radiolabeling

For [^35^S] labeling, transfected 293T cells were preincubated in Met/Cys-free media (Sigma), labeled for 45 min with 0.4 mCi/mL [^35^S] Met/Cys (MP Biomedical) and chased for 3 h in complete RPMI-medium containing 100 μg/mL methionine and 500 μg/mL cysteine. For [^32^P] orthophosphate labeling, transfected 293T cells were incubated for 30 min at 37°C in phosphate-free MEM containing 10% dialyzed FCS and for another 4 h in the same medium supplemented with 0.5 mCi/mL [^32^P] orthophosphate (Perkin Elmer). Culture medium was removed and cells were washed extensively and then lysed in buffer containing 1% Triton-X-100. Radiolabeled cystatin F from culture medium and lysates were immunoprecipitated using cystatin F antibodies (FL Ab) as described (16). Aliquots of immunoprecipitated cystatin F were left untreated or digested with EndoH as described (16). For ^125^I-labeling experiments, 20 μg of purified recombinant cystatin F was labeled with 0.5 mCi [^125^I]-Na (Amersham) using the IODO-GEN reagent (Pierce) following manufacturer’s instructions.

### Internalization assay

To follow the internalization of cystatin F, L929 cells were cultured at 5 × 10^5^/mL in 10-cm culture dishes or 2 × 10^5^ on glass coverslips for 24 h followed by the addition of normalized cystatin F supernatants taken from 293T cultures for an additional 24–48 h. The cells were then washed twice in 100 mmglycine, pH 3.0, lysed and analyzed for cystatin F internalization or cathepsin activity as described below. For studies using [^125^I]-labeled cystatin F, L929 cells were incubated with 500 μg/mL [^125^I] cystatin F in the presence or absence of 10 mmM6P at 4°C. The cells were washed three times with PBS to remove unbound protein and incubated at 37°C for 30 min to allow for internalization. Before lysis, cystatin F remaining on the plasma membrane was removed by acid stripping (100 mmglycine, pH 3.0). Control cells were maintained at 0°C throughout. Percent internalization was determined by dividing the acid stable fraction following acid washing at 30 min from the acid labile fraction at 0 min. Primary murine CD8^+^ T lymphocytes (CTL) and dendritic cells were isolated from the spleen and bone marrow of C57Bl/6 mice as described (16,47). Primary CD8^+^ splenocytes from CD45.2^+^, cystatin F knockout mice or CD45.1^+^, cystatin F-expressing mice were either cultured independently or at a 1:1 ratio for 48 h. Surface co-labeling of CD45.1 (fluorescein isothiocyanate) and CD45.2 (APC) were detected by flow cytometry using directly conjugated antibodies. Internalization of cystatin F was determined by surface staining of either CD45.1 (APC) or CD45.2 (APC) followed by intracellular staining for cystatin F (FL Ab) using the Fix & Perm cell permeabilization kit (Caltag Laboratories).

### Microscopy

Transfected 293T cells were grown on poly-l-lysine-coated coverslips for 48 h before staining. Cells were then fixed with 4% paraformaldehyde, permeabilized with 0.2% Triton-X-100 and costained with rabbit anti-cystatin F (FL Ab), and either mouse anti-CD63 or sheep anti-TGN46 in the presence of 1% (w/v) BSA as described (16). For internalization assays, L929 cells were grown on poly-l-lysine-coated coverslips for 18 h and treated with cystatin F-containing conditioned media or control media as described above. Primary antibodies were detected using Alexa-488 or Alexa-594-conjugated anti-rabbit (Invitrogen), TR-conjugated anti-sheep (pseudo-colored green) (Jackson Laboratory) and Alexa-488 anti-mouse (Invitrogen) secondary antibodies. Stained cells were mounted on glass slides and imaged on a LSM 510 META confocal microscope using a 100× PlanFluar/NA 1.45 objective and lsm510 software (Carl Zeiss Inc.) followed by processing with Photoshop (Adobe).

## Supporting Information

### Figure S1: Cystatin F inhibitory activity depends on proper subcellular localization

293T cells were left alone or transfected with either wild-type cystatin F or various glycosylation mutants. Cathepsin C activity was determined using 10 μg of total protein from cell lysates. Data represent the mean rate of cathpesin C activity from two independent experiments.

### Figure S2: Internalization of cystatin F is not inhibited in the presence of free mannose or fucose

Equal concentrations of secreted wild-type cystatin F produced by 293T cells were cultured with L929 cells for 48 h in the absence (untreated) or presence of 10 mM of the free sugars indicated. L929 cell lysates were separated by SDS–PAGE under nonreducing conditions and blots stained for internalized cystatin F (Cyst. F)

### Figure S3: Amino acid sequence alignment of cystatin F N-terminal glycosylation motifs

Partial cystatin F sequence alignments were performed using Geneious Basic (Biomatters Ltd) as described. Amino acids 50–72 and 105–130 (human numbering) are displayed. Nonconsensus N-linked glycosylation motifs (NXC) are shown in red, while consensus sequences (NXS/T) are shown in blue. The sequence for *Danio rerio* was found to be incomplete.

### Figure S4: Internalization and activation of exogenously added cystatin F in cystatin F-deficient splenocytes

A) Knockout splenocytes were treated either with a medium control (white fill) or with 5 μm of recombinant cystatin F (gray fill) for 48 h followed by intracellular cystatin F staining by FACS analysis, (B) intracellular cystatin F staining by confocal microscopy (green punctate stain) or (C) cathepsin C activity in postnuclear cell lysates. Data are from protein equivalents and are the mean rate from two independent experiments. Bars, 10 μm.
