# Supplementary material for: Glycosylation Directs Targeting and Activation of Cystatin F from Intracellular and Extracellular Sources
Source: Traffic. 2009 Apr;10(4):425–37. doi: 10.1111/j.1600-0854.2009.00881.x (PMC2691902; doi:10.1111/j.1600-0854.2009.00881.x)
Supplement: Supplementary file 3 [file tra0010-0425-SD3.pdf]

Organism	Species	Amino Acid Sequence
		50 60 70 110 120 130
Human	*Homo sapiens*	...LQAARYSVEKFNNCTNDMFLFKE...LRLDDCDFQTNHTLKQTLSCYSEVWV...
Rhesus Monkey	*Macaca mulatta*	...LQAARHSVEKFNNCTNDMFLFKE...PRLDDCDFQTNQTLKRTLSCYSEVWV...
Dog	*Canis familiaris*	...LQAARHSVERFNNCTNDIFLFKE...PSLDNCDFQTNRTLKQTFSCYTEVWV...
Cow	*Bos taurus*	...LRAARHSAESFNNCSNDAFLFRE...ANLDDCSFQTNRTLQWTLSCYSEVWV...
Horse	*Equus caballus*	...LKAARHSVERFNNCTNDIFLFKE...PSLDNCDFQTNSTLKRTLSCYSEVWV...
Pig	*Sus scrofa*	...LRAARHSAESFNNCSNDAFLFKE...ASLDNCSFQTNHSLQWTFSCYSEVWV...
Mouse	*Mus musculus*	...LKAARHSVEKFNNCTNDIFLFKE...HQLDNCDFQTNPALKRTLYCYSEVWV...
Rat	*Rattus norvegicus*	...LKAARHSVEKFNNCTNDIFLFKE...RQLDNCDFQTSPALKRTLHCYSEVWV...
Opossum	*Monodelphis domestica*	...QKAARFAVERFNNCTNDLFLFKE...SNLDNCDFQTDITLKQTFSCYTEVWV...
Red Jungle Fowl	*Gallus gallus*	...RKAARFGVYQFNNSSNDLFLFKE...SNLDDCHFQKKKNLQQILKCYFEVWM...
Clawed Frog	*Xenopus tropicalis*	...KNAARVTVYAYNNKSNDSFLFKE...YNLDQCDFQEDNPLKQTFSCYSEVWN...
Zebrafish	*Danio rerio*	...KKAVLTGTYSFNNKSNDAFLFKA...EDLINCPFQTDSLLRQ----------

Figure S3
